# Peripheral substance P induces deficits in hippocampal synaptic plasticity and memory

**DOI:** 10.1186/s13041-025-01242-6

**Published:** 2025-08-16

**Authors:** Sun Yong Kim, Kyeong-No Yoon, Jungeun Ji, Min-Gyun Kim, Seung Ah Choi, Gunhyuk Park, Won-Woo Lee, Jin Ho Chung, Sang Jeong Kim, Joon-Yong An, Dong Hun Lee, Yong-Seok Lee

**Affiliations:** 1 Department of Physiology, Seoul National University College of Medicine, Seoul, 03080 Republic of Korea; 2 Department of Biomedical Sciences, Seoul National University College of Medicine, Seoul, 03080 Republic of Korea; 3 Department of Dermatology, Seoul National University College of Medicine, Seoul National University Hospital, Seoul, 03080 Republic of Korea; 4 Department of Integrated Biomedical and Life Science, Korea University, Seoul, 02841 Republic of Korea; 5 Herbal Medicine Resources Research Center, Korea Institute of Oriental Medicine, 111 Geonjae-ro, Naju, 58245 Republic of Korea; 6 Institute of Human-Environment Interface Biology, Seoul National University, Seoul, 03080 Republic of Korea; 7 Neuroscience Research Institute, Seoul National University College of Medicine, Seoul, 03080 Republic of Korea; 8 Wide River Institute of Immunology, Seoul National University, Hongcheon, Republic of Korea

**Keywords:** Substance P, Synaptic plasticity, Hippocampus, Long-term potentiation

## Abstract

Substance P (SP) is a neuropeptide that functions in both the central and peripheral nervous systems. Although the peripheral actions of SP in regulating inflammatory responses have been extensively investigated, the effects of elevated peripheral SP on hippocampal functions such as spatial learning and memory remains unclear, even though SP can cross the blood-brain barrier. In this study, we found that male mice subcutaneously injected with SP for 14 days exhibited significant deficits in hippocampus-dependent memory, as assessed by the object place recognition and novel object recognition tests. In addition, long-term potentiation (LTP) at the hippocampal CA3–CA1 synapse was reduced in SP-treated mice. Transcriptomic analyses identified 77 differentially expressed genes (DEGs), and enrichment analysis highlighted pathways related to synaptic transmission, learning, and memory. These results suggest a novel skin–brain neuropeptide signaling axis. Targeting peripheral SP or its receptor may provide a therapeutic avenue for cognitive dysfunction associated with peripheral inflammation.

**Supplementary Information:**

The online version contains supplementary material available at 10.1186/s13041-025-01242-6.

## Introduction

Substance P (SP) is a neuropeptide that functions in both the central and peripheral nervous systems [1, 2]. It is widely distributed in the brain and peripheral organs, with high concentrations in the intestine and skin [3]. In the periphery, SP-containing neurons convey nociceptive signals to the spinal cord, highlighting their roles in pain processing [4–6]. Additionally, SP is critically involved in neurogenic inflammation. For example, SP mediates ultraviolet (UV) irradiation-induced skin inflammation [7]. It was shown that SP-immunoreactive fibers in the skin increase 24 h after UV exposure [8]. Furthermore, SP contributes to itching and inflammation through the activation of neurokinin 1 receptors (NK-1R) in peripheral regions [5, 9] and may influence the balance of cytokines [6].

In addition to its peripheral actions, SP is abundantly present in the central nervous system. SP-containing axon terminals are distributed in the hippocampus, including the CA2 region, stratum oriens of CA1/CA3, and subiculum [10]. Furthermore, SP receptors, particularly NK-1R, are widely expressed in these hippocampal regions [11–13]. Because the hippocampus is critical for spatial learning and memory [14], several studies have investigated the effects of SP on cognitive functions. For instance, blocking NK-1R mRNA in either the hippocampus or striatal marginal division via antisense oligonucleotides impaired spatial learning and memory in rats [15]. Conversely, acute intraperitoneal injection of SP improves water maze performance [16], or facilitates retention test performance when administered after training in rats [17]. These findings suggest that SP signaling in the brain can modulate cognitive processes, although the effects may vary depending on the context, duration, and site of SP exposure.

The skin is not only a barrier and sensory organ but also a large endocrine organ [18]. Recent studies have highlighted skin-brain interactions in modulating brain function [19–21]. SP, produced and released from cutaneous sensory nerve endings [22–24], can enter the systemic circulation and, due to its permeability across the blood-brain barrier, potentially influence central nervous system function [25]. The widespread distribution of NK-1R in the brain, including key hippocampal subregions [11–13], further supports the plausibility of a skin–brain neuropeptide axis mediated by SP. However, the impact of chronic peripheral elevation of SP-such as might occur during persistent skin inflammation-on hippocampal function and cognition remains poorly understood. Here, we hypothesize that chronic elevation of peripheral SP, as observed in conditions such as UV-induced skin aging, can affect hippocampus-dependent memory and synaptic plasticity. Although SP is traditionally recognized for its role in peripheral nociceptive signaling, its ability to cross the blood–brain barrier raises the possibility that sustained increases in peripheral SP may modulate central neural circuits. Therefore, our study was designed to test the hypothesis that persistent peripheral SP elevation impacts hippocampal function, potentially contributing to cognitive impairment through a novel skin–brain neuropeptide signaling axis.

## Materials and methods

### Mice

Male C57BL/6 N mice were purchased from Orient Bio (South Korea) and used for experiments after at least 1 week of adaptation. Eight-to-ten-week-old mice were used in the experiments. Mice were housed under a 12-hour light/dark cycle (lights on: 8:00–20:00) with food and water *ad libitum.*

### Administration of substance P

SP was dissolved in distilled water and stored at -20 °C before use. Substance P (10 µM, 100 µL) was injected subcutaneously (s.c.) for 14 consecutive days. The injection dosage was based on a previous study, which has been shown to induce neutrophil infiltration and mast cell degranulation in mouse skin [26]. The same volume of saline was injected subcutaneously into control mice.

### Behavioral tests

Behavioral tests were performed as described previously with slight modifications [27–29]. In object place recognition (OPR) test, mice were handled for 5 min for 4 days before performing the experiment. For two consecutive days, mice were habituated in a square acrylic box (32 cm × 32 cm × 32 cm) for 15 min, and a local cue (star-shaped) was placed on one side of the box. On the training day, the mice were placed in an acryl box with two identical glass bottles for 10 min to freely wander and explore the objects. Twenty-four hours after training, the mice were exposed to the same square box with the object in the same location and the other object that was moved to a new location. In novel object recognition (NOR) test, one of the objects used on the training day was replaced with a novel object on the test day. All object locations were counterbalanced among the groups, and the box with objects was cleaned between trials. Sessions were videotaped and later analyzed manually while the experimenter was blinded to the experimental groups.

### Electrophysiology

Extracellular field excitatory postsynaptic potential (fEPSP) recordings were performed as previously described [30]. Briefly, hippocampal sagittal slices (400 μm) were prepared by using a vibratome (Campden Instruments, Oxford, UK) in ice-cold artificial cerebrospinal fluid (ACSF; 120 mM NaCl, 3.5 mM KCl, 2.5 mM CaCl_2_, 1.3 mM MgSO₄, 1.25 mM NaH_2_PO_4_, 10 mM glucose, and 26 mM NaHCO₃, oxygenated with 95% O_2_ and 5% CO_2_). The fEPSPs from the Schaffer collateral-CA1 pathway were recorded at 31–32 °C. A stimulation intensity of 40% of the maximum response was selected for this experiment. Input-output relationships were measured by giving 0-100 µA of stimulation intensities and paired pulse ratios by measuring fEPSPs at different time intervals (10, 25, 50, 100, 200, and 400 ms). The theta burst stimulation protocol (four bursts, where each burst consisted of four pulses at 100 Hz and 200-ms inter-burst intervals) was used to induce long-term potentiation (LTP). WinLTP software (WinLTP Ltd., Bristol, UK) was used to record and analyze the data.

### RNA sequencing and analysis

Hippocampi from 3 SP-injected and 3 vehicle-injected mice were analyzed. Read alignment and processing were performed using the Nextflow RNAseq pipeline [31]. Briefly, raw reads were trimmed with TrimGalore (https://github.com/FelixKrueger/TrimGalore), aligned against the mouse reference genome GRCm38 using STAR [32], and quantified with Salmon [33]. Differential gene expression analysis was performed using R package DESeq2 (v1.42.1) [34], adjusting for replicates. Genes with FDR < 0.1 and |log₂FC| >0.3 were considered significant differentially expressed genes (DEGs). Gene Ontology (GO) biological processes enriched for significant DEGs were identified using clusterProfiler (v 4.10.0) [35].

### Statistics

For behavior data such as OPR test and NOR test, a paired two-tailed t-test was used to compare the exploration time (%) of novel object and old object in the same group of mice. LTP data were analyzed using a 2-way ANOVA, and two-tailed unpaired t-test on averaged data collected in the last 10 min of recording. Data normality was assessed by using the Shapiro-Wilk test prior to statistical analysis. For the immunohistochemical analysis of DCX-positive cells, the non-parametric Mann-Whitney U test was applied without a prior normality test due to the small sample size. GraphPad Prism version 9.5.1 (GraphPad Software, San Diego, CA, USA) was used. All data are presented as mean ± SEM.

## Results

### Chronic substance P injection impairs hippocampal learning and memory

**Fig. 1:**
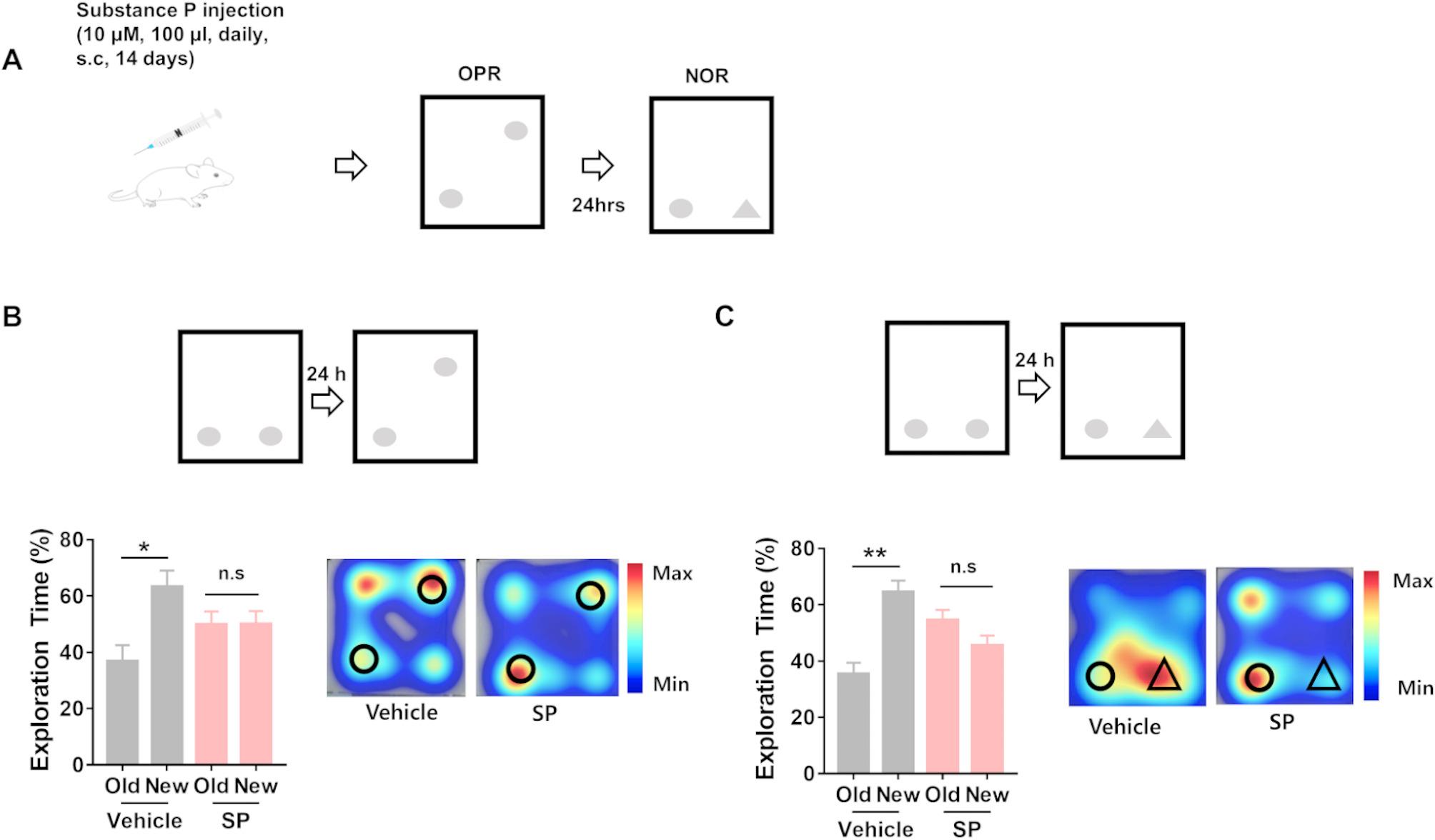
Chronic substance P injection impairs hippocampal learning and memory. (**A**) Experimental scheme for substance P (SP) injection and behavior. Mice were subcutaneously injected with SP (10µM, 100 µL) for consecutive 14 days. The control group was injected with vehicle (saline). Two weeks after the injection, object place recognition (OPR) and novel object recognition (NOR) tests were performed. (**B**) Experimental scheme for the OPR test. In the OPR test, the mice were exposed to two identical objects in a square box for training, and one object was moved to a different location after 24 h. While vehicle group mice (*n* = 13) could differentiate objects moved from the object in the previous place, SP-injected mice (*n* = 15) could not. Paired two-tailed t-test, * *p* = 0.0408 (old vs. new in control group), *p* = 0.9855 (old vs. new in SP group) Representative heat maps of two groups are shown. (**C**) Experimental scheme for NOR test. In the NOR test, mice were exposed to two identical objects during the training session, and one object was replaced with a new object after 24 h. SP-injected mice could not discriminate between a novel object and a familiar object. Paired two-tailed t-test, ** *p* = 0.003 (old vs. new in control group, *n* = 13 mice), *p* = 0.22 (old vs. new in SP group, *n* = 15 mice). Representative heat maps of the vehicle and SP groups are shown.

To investigate the effects of chronic peripheral SP treatment on hippocampal learning and memory, we injected SP (10 µM, 100 µL) subcutaneously into wild-type male mice for 14 consecutive days. No visible inflammation or histopathological abnormalities were observed in SP-injected mice compared to saline-injected control mice (Supplementary Fig. 1). After 14 days of SP injection, the mice were subjected to the OPR and NOR test which are hippocampus-dependent behavioral tasks (Fig. 1A). In the OPR test, substance P-injected mice did not show a preference for a moved object over an object in a familiar location, while control mice injected with saline spent significantly more time exploring the moved object (Fig. 1B). In addition, SP-injected mice did not differentiate the familiar object from the new object in the NOR test, unlike the vehicle group which spent significantly more time exploring the novel object than the familiar object (Fig. 1C). Collectively, these results show that chronic peripheral SP treatment impairs hippocampus-dependent memory.

### Chronic peripheral substance P induces hippocampal LTP deficit

**Fig. 2:**
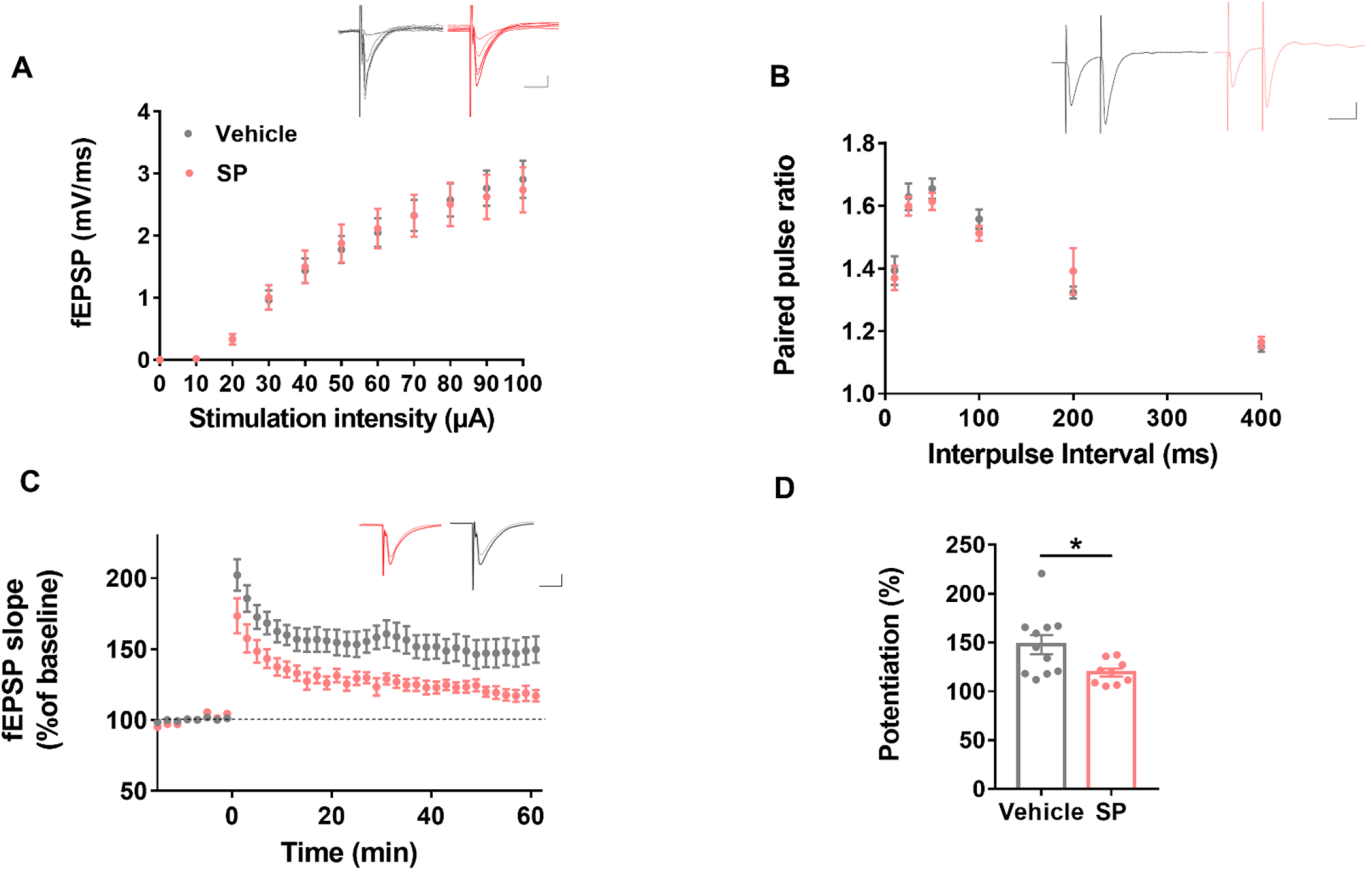
Peripheral substance P induces hippocampal LTP deficit. (**A**) Input-output curves of fEPSP slope in response to increasing stimulus input (0-100 µA) were similar between the substance P (SP) (*n* = 16 slices from 6 mice) and vehicle groups (*n* = 16 from 7 mice). *p* = 0.976 by 2-way ANOVA. Black traces represent input-output curves from the vehicle group. Red traces represent input-output curves from the SP group. (**B**) Paired pulse ratio was comparable between the SP (*n* = 16 slices from 6 mice) and vehicle groups (*n* = 16 slices from 7 mice). *p* = 0.76, 2-way ANOVA. Black traces represent fEPSP traces from the vehicle group and red traces represent the SP group. (**C**) Time course of fEPSP slope. LTP was induced by 4X TBS (4X theta burst stimulation, each burst with four stimuli at 100 Hz, 200 ms inter-burst interval). The SP-injected group (*n* = 9 slices from six mice) had significant LTP deficits compared to the vehicle group (*n* = 11 slices from seven mice). The fEPSP slopes were normalized to the average baseline values. Black traces represent fEPSP traces from the vehicle group and red traces represent fEPSP slopes from the SP group. (**D**) The average fEPSP slope 51–60 min after LTP induction. The SP-injected group (*n* = 9 slices from six mice) showed LTP deficits in the last 10 min compared to the vehicle group (*n* = 11 slices from seven mice). A significant difference: *p = 0.0225 by unpaired two-tailed t-test. Vertical bar, 0.5 mV; horizontal bar, 10 ms. Error bars represent mean ± SEM.

LTP is a cellular mechanism that underlies spatial learning and memory in the hippocampus [14]. We examined whether peripheral SP injection causes deficits in hippocampal LTP by recording fEPSP at the CA3-CA1 Schaffer collateral pathway. Chronic peripheral SP treatment did not have significant effects on the input-output relationship or paired pulse facilitation ratio (PPF) compared to the saline-injected control group (Fig. 2A and B), indicating that basal synaptic transmission or short-term plasticity was not affected. However, the SP-injected group showed significant hippocampal CA3-CA1 LTP deficits compared to the controls (Fig. 2C and D). These findings demonstrate that peripheral administration of SP impairs CA3-CA1 hippocampal LTP, which may underlie the observed memory deficits.

### Chronic peripheral substance P impairs adult neurogenesis in the hippocampus

**Fig. 3:**
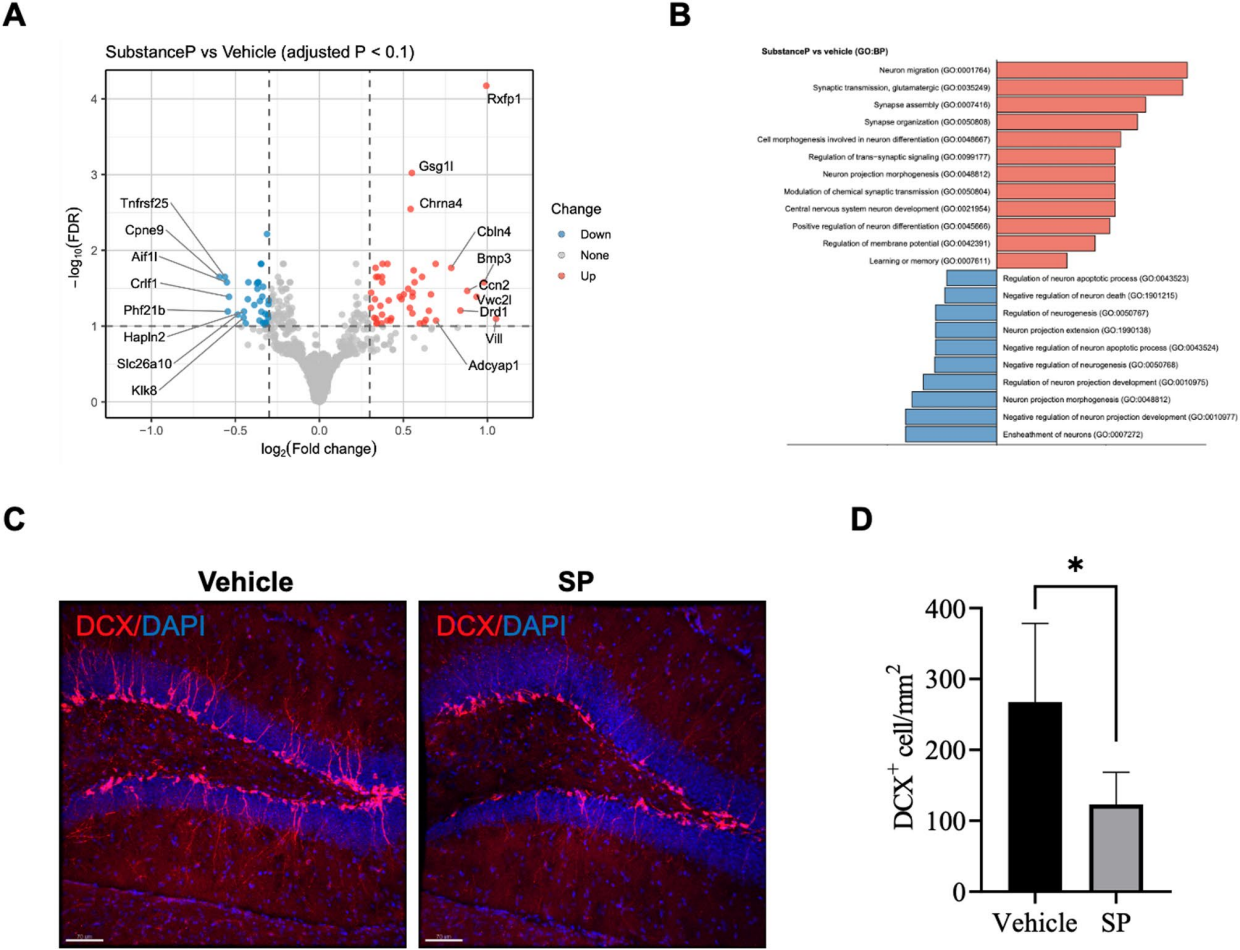
Transcriptomic and histological alterations following peripheral substance P injection. (**A**) Volcano plot showing differentially expressed genes (DEGs) identified by RNA-Seq in the brains of substance P (SP)-injected mice compared with controls (FDR < 0.1, |log₂FC| >0.3). Upregulated genes are indicated in red, downregulated genes in blue, and nonsignificant genes in gray. (**B**) Gene ontology (GO) enrichment analysis of upregulated and downregulated DEGs, highlighting the enriched biological processes associated with synaptic transmission, membrane potential regulation, learning/memory, and neurogenesis. (**C**) Representative immunohistochemical images showing DCX expression in the hippocampal dentate gyrus of control and SP-injected mice. Scale bar = 70 μm. (**D**) Quantification of DCX-positive cells showing a significant reduction in DCX expression following SP injection. Data are presented as mean ± SEM; *p < 0.05, versus control (*n* = 4 per group, Mann–Whitney U test).

To gain molecular insight into how the chronic peripheral exposure to SP impairs memory and synaptic plasticity, we performed bulk RNA sequencing (RNA-seq) analysis. RNA‑Seq identified 77 DEGs in the hippocampi of SP animals compared to controls at FDR < 0.1 and |log₂FC| >0.3, comprising 44 upregulated and 33 downregulated transcripts (Fig. 3A, Supplementary Table 1). Of particular interest, upregulated DEGs, such as *Gsg1l* (a negative regulator of AMPAR‑mediated transmission) [36] and *Cbln4* (a synaptic adaptor essential for spatial memory) [37], suggest perturbed α-amino-3-hydroxy-5-methyl-4-isoxazolepropionic acid receptor (AMPAR) homeostasis and cerebellin‑mediated circuit modulation. The GO enrichment analysis revealed that up‑regulated genes clustered in synaptic transmission and membrane potential pathways (*Drd1*,* Adcyap1*,* Cckbr*,* Reln*,* Htr1b*,* Mef2c*) and learning/memory processes (*Drd1*,* Reln*,* Mef2c*,* Nr4a2*), whereas downregulated genes included *Mag*,* Mbp*, and *Sema5b*, which are known to participate in oligodendrocyte maturation, myelination, and axonal guidance [38]. Although these genes have been previously suggested to act as negative regulators of neurogenesis, accumulating evidence indicates that they are more broadly involved in the maturation and maintenance of the neurogenic microenvironment, including the regulation of synaptic connectivity and glial support [39–41]. Their downregulation may thus reflect impaired structural support for neurogenesis rather than the facilitation of stem cell activity (Fig. 3B, Supplementary Tables 2 and 3).

Consistent with the transcriptomic findings, previous studies have also shown that UV irradiation which induces skin inflammation in mouse skin decreases hippocampal neurogenesis [20, 29]. To validate decreases in neurogenesis in the hippocampus, we performed immunohistochemical staining for doublecortin (DCX), a marker of neurogenesis, and observed a significant reduction in DCX expression in the hippocampus of SP-injected mice compared to controls (Fig. 3C and D). Although DCX reflects the presence of immature neurons, it does not mark proliferating cells. Therefore, it may indicate reduced neuronal maturation rather than definitively confirming a reduction in progenitor proliferation.

## Discussion

SP levels are elevated in chronic inflammatory skin conditions including psoriasis [42] and atopic dermatitis [43], and various external stressors, particularly UV irradiation, can enhance peripheral SP expression [8]. These findings suggest that chronic inflammatory skin diseases may adversely affect hippocampal function, potentially mediated through elevated peripheral SP signaling. Although previous studies have addressed the function of SP in the central nervous system, particularly in stress mechanisms and mood regulation [44–46], the impact of chronic peripheral SP on hippocampal LTP and memory has not been thoroughly studied. In guinea pig hippocampal slices, acute SP treatment increased baseline fEPSP slopes and significantly increased synaptic potentiation via NK-1R activation [47]. In contrast, we found that chronic SP treatment via subcutaneous injection impaired hippocampal LTP without affecting the basal fEPSP slope in mice. These discrepant results may be attributed to the different durations of SP exposure. While the previous study applied SP only during slice recording [47], we administered it chronically for two weeks in vivo. In addition, differences in experimental species and administration methods may also have contributed to these different outcomes.

Consistent with these behavioral and electrophysiological deficits, hippocampal RNA-seq revealed significant dysregulation of genes related to synaptic transmission, neurogenesis, and dopamine signaling. DEGs such as the synaptic‑transmission gene *Gsg1l* and the synaptic adaptor *Cbln4* further implicate disrupted AMPAR regulation and spatial-memory circuitry in the observed phenotypes. In addition, our RNA-seq analysis revealed downregulation of genes such as *Mbp*, *Mag*, and *Sema5b*, which are involved in glial differentiation, myelination, and neuronal integration [48]. While these genes are not canonical markers of neurogenesis, their reduced expression may indicate broader neurodevelopmental and neuroplastic changes that compromise the neurogenic microenvironment. These alterations may indirectly contribute to the observed suppression of adult hippocampal neurogenesis. Furthermore, given that DCX labels immature neurons but not proliferating progenitor cells, future studies incorporating additional markers such as Ki-67 or BrdU will be necessary to more comprehensively characterize changes in adult hippocampal neurogenesis. Reduced neurogenesis has been implicated in memory deficits and impaired hippocampal plasticity [29, 49]. Interestingly, although *Mag*, *Mbp*, and *Sema5b* are sometimes referenced as negative regulators of neurogenesis, their primary roles lie in glial differentiation, myelination, and axon guidance [48]. The observed reduction in their expression may reflect a compromised neurogenic niche or disrupted neural maturation processes rather than promoting neurogenesis per se. This interpretation aligns with the reduced DCX expression observed in SP-injected mice, suggesting an overall suppression of adult hippocampal neurogenesis.

Notably, we found that *Drd1*, a dopamine D1 receptor gene critical for hippocampal LTP and memory consolidation, was markedly upregulated in the hippocampus of SP-exposed mice. This observation raises the possibility that altered dopaminergic signaling may contribute to memory impairments. Although a previous study has suggested that chronic peripheral stressors, such as UV exposure, can modulate dopaminergic pathways [29], further experimental validation is required to determine whether *Drd1* plays a causal role in SP-induced hippocampal dysfunction. Together, these results suggest that peripheral stressors, whether inflammatory or environmental, may converge on dopaminergic signaling pathways to disrupt hippocampal function.

A major limitation of the present study is the lack of pharmacological or genetic interventions targeting the SP–NK1R signaling axis. Although our findings implicate peripheral SP in hippocampal dysfunction, definitive causal inference will require future studies employing NK1R antagonists or genetically engineered models with selective disruption of this pathway. It is worthy to note that our histological analyses did not reveal overt inflammatory changes in the skin at the injection sites (Supplementary Fig. S1), although SP and its receptor NK-1R are known to participate in neuroinflammatory processes [50]. Given this, our findings suggest a model in which chronic peripheral SP elevation disrupts hippocampal signaling pathways including dopaminergic and neurogenic pathways, leading to impaired synaptic plasticity and memory. However, we cannot fully exclude the possibility of subclinical immune activation, which may not be apparent through conventional histological evaluation. Future studies assessing peripheral cytokine levels, such as IL-1β and TNF-α, as well as mast cell degranulation, would be valuable in clarifying whether immune-related mechanisms contribute to the observed hippocampal deficits.

Although peripheral SP has been shown to cross the blood-brain barrier via a carrier-mediated nonendocytic mechanism [25], our study does not provide direct evidence of SP accumulation in the brain. Therefore, we cannot exclude the possibility that the observed hippocampal effects may be mediated indirectly through peripheral immune responses, systemic cytokine signaling, or hypothalamic–pituitary–adrenal (HPA) axis activation. Future studies using labeled SP tracers or BBB permeability assays will be essential to clarify whether SP directly reaches the hippocampus or exerts its effects via alternative systemic pathways. Accordingly, we speculate that excessive peripheral SP can directly modulate hippocampal synaptic plasticity. However, future studies are needed to clarify whether peripheral SP directly reaches the brain to cause LTP deficits or exerts its effects via other systemic mechanisms.

In conclusion, our findings provide evidence that excessive peripheral SP impairs hippocampal memory and synaptic plasticity. This study not only reveals a novel role of peripheral SP in hippocampal function but also offers insight into the mechanisms underlying skin-brain interactions. These findings expand the understanding of peripheral neuropeptide-mediated modulation of central nervous system functions, and highlight potential therapeutic targets for cognitive deficits associated with peripheral stressors.

## Supplementary Information

Below is the link to the electronic supplementary material.

Supplementary Material 1: List of differentially expressed genes in the hippocampus of SP-treated mice

Supplementary Material 2: Gene Ontology pathway of up-regulated DEGs

Supplementary Material 3: Gene Ontology pathway of down-regulated DEGs

Supplementary Material 4

## Data Availability

The datasets generated and analyzed during the current study are available in the NCBI Gene Expression Omnibus (GEO) under accession number GSE301993. Further inquiries can be directed to the corresponding authors.

## Abbreviations

AMPAR: α-amino-3-hydroxy-5-methyl-4-isoxazolepropionic acid receptor
ANOVA: analysis of variance
DCX: doublecortin
DEG: differentially expressed genes
FDR: false discovery rate
fEPSP: field excitatory postsynaptic potential
GO: gene ontology
LTP: long-term potentiation
NK-1R: neurokinin 1 receptor
NOR: novel object recognition
OPR: object place recognition
PPF: paired pulse facilitation ratio
SP: substance P
UV: ultraviolet
