# Supplementary material for: Peripheral substance P induces deficits in hippocampal synaptic plasticity and memory
Source: Mol Brain. 2025 Aug 16;18:69. doi: 10.1186/s13041-025-01242-6 (PMC12358064; doi:10.1186/s13041-025-01242-6)
Supplement: Supplementary file 4 — Supplementary Material 4 [file 13041_2025_1242_MOESM4_ESM.docx]

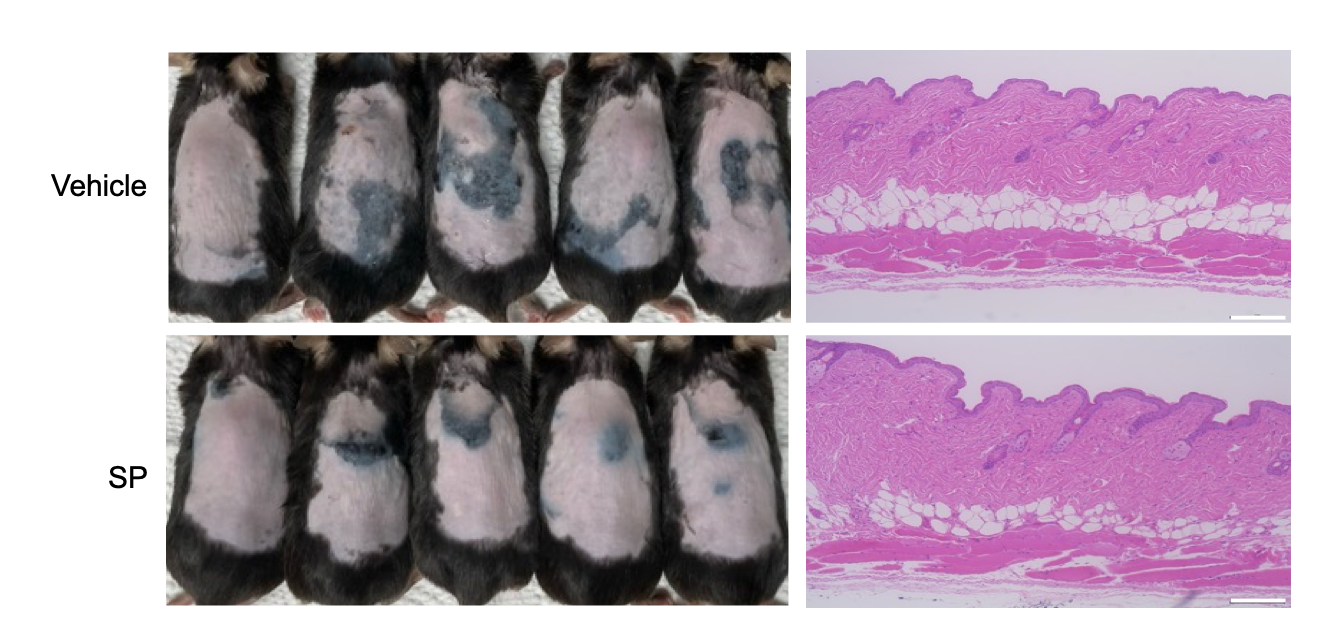


**Supplementary Figure. S1.**

Representative gross images and H&E-stained sections of the skin at the injection site 14 days after subcutaneous substance P injection, showing no visible inflammation or histopathological abnormalities compared to saline-injected controls. Scale bar = 10 μm.
